# Agents’ awareness of visuo-motor incongruency determines changes in haptic sensitivity

**DOI:** 10.1007/s00221-026-07309-7

**Published:** 2026-05-06

**Authors:** Yanick Kloss, Wilfried Kunde

**Affiliations:** Department of Psychology (III), University of Würzburg, Würzburg, Germany

**Keywords:** Tactile sensitivity, Visuo-motor incongruency, Action control, Ideomotor theory

## Abstract

When body movements are transformed into somewhat discrepant effects in the environment, agents are surprisingly unaware of what their body is doing exactly (e.g., Knoblich and Kircher 2004; Fourneret and Jeannerod 1998; Sutter et al. 2008). Presumably, that is because agents control such movements primarily via their transformed visual effects while downregulating the processing of haptic movement effects (for a review, see Sutter et al. 2013). In two experiments, we measured tactile sensitivity on the moving effector as a proxy for the up- or downregulation of processing haptic effects during continuous visuo-motor congruent or incongruent tool transformations. As expected, tactile sensitivity was modulated by incongruency, but only when individuals became aware of it. Awareness came with an increase in tactile sensitivity, suggesting that haptic processing is upregulated once agents notice that their movements are not congruent with intended visual effects. These findings challenge the presumed dominance of environment-related action consequences in movement control, demonstrating the breakthrough of tactile perception in case of visuo-haptic incongruency.

## Introduction

We typically move our bodies to produce all kinds of perceptual changes in the environment.

When using tools, these movements are often transformed into somewhat discrepant effects, and we need to adjust movements accordingly. Consider operating your computer mouse: a small rightwards mouse movement will produce a much larger visual cursor movement on the screen. While we can obviously adapt to such incongruent tool transformations, they remain challenging ((Heuer and Hegele 2008) Hegele and 2010). If you were to increase the speed of your cursor, for instance, you may experience difficulties adjusting. And if you went one step further and turned the mouse upside down, you would have a really hard time clicking on the file in the top right corner by moving your hand to the bottom left (Kunde et al. 2007).

One reason behind limitations to control incongruent visuomotor transformations may lie in the diverse nature of the perceptual effects our motor activities produce. In fact, in neurotypical agents they are never limited to effects originating from the environment. Instead, agents will experience proprioceptive or tactile (or shortly haptic) changes in and around the moving effector as well (Pfister 2019). Frameworks inspired by ideomotor theory propose that actions are represented in terms of these various effects ((Shin et al. 2010) for a review). When they are incongruent to one another, e.g., in terms of spatial direction (e.g., (Chen and Proctor 2013; Pfister and Kunde 2013)), time-point (Dignath et al. 2014), or intensity (Kunde et al. 2004), they can interfere and impair performance.

There are several lines of evidence suggesting that in such instances of *code interference* (Janczyk and Kunde 2020), agents tend to control their actions primarily via their visual effects, while downregulating the processing of body-related effect codes, a phenomenon referred to as *functional haptic neglect* (for a review, see (Sutter et al. 2013)). First, agents often show remarkably little insight into how their body moves to achieve a certain visual effect. When asked to reproduce their movements their reproductions tend to shift strongly toward the visual effects (Debats and Heuer 2018; Debats et al. 2017; Heuer and Rapp 2012; Knoblich and Kircher 2004; Ladwig et al. 2012; Müsseler and Sutter 2009; Sutter et al. 2008, 2013). Second, stimulus–response (S-R) compatibility effects seem to primarily emerge between stimulus features and features of the response’s effects in the environment, rather than at the body (Kunde et al. 2007; Müsseler et al. 2008; Proctor et al. 2004; Sabek et al. 2025). Finally, a deafferent patient without any bodily perception showed no performance cost with incongruent visual movement effects (Lajoie et al. 1992), strongly suggesting that body-related movement effects are a driver of such costs in neurotypical agents.

However, the dominance of environment-related effects for action control is not set in stone. Instead, individuals appear capable of dynamically up- and downregulating the processing of different effect modalities (Heuer and Rapp 2012; Thébault et al. 2020; Wirth et al. 2016). For instance, increasing the task relevance of bodily effects can reverse S-R compatibility effects that were previously driven by the effects in the environment (Sutter and Ladwig 2012), presumably due to an upregulation of body-related codes. Yet, when the reliability of proprioceptive feedback is reduced, the original pattern re-emerges—suggesting that actions are once again represented primarily via their environmental consequences.

Here, we investigated a factor overlooked in many studies of visuo-motor incongruency: individuals’ awareness of the mismatch between their movement and its effects in the environment (Rand and Heuer 2018; Sülzenbrück and Heuer 2009; Sutter et al. 2013). In conditions in which visuo-haptic movement consequences are obviously incongruent, such that a leftwards hand movement produces a rightwards cursor movement (e.g., (Heuer and Rapp 2012; Kloss and Kunde 2026; Kunde et al. 2007, 2012; Wendker et al. 2014)), individuals arguably become aware of this obvious incongruency and might exert top-down control over their mode of action control (Sülzenbrück and Heuer 2009). They might deliberately focus more (and not less) on what their body needs to be doing while perhaps even ignoring the visual effects as best as possible, a strategy frequently reported by our participants (Kloss and Kunde 2026).

There are paradigms in which the incongruency between haptics and vision is more subtle, and individuals in fact often do not become aware of it at all (e.g., (Fourneret and Jeannerod 1998; Kirsch et al. 2016; Rand and Heuer 2013; Sack and Sutter 2017; Sutter et al. 2008; Wang et al. 2012), [60]). In a typical paradigm, participants produce movements that are transformed into slightly discrepant visual effects. When later asked to judge characteristics of their movement, such as the overall distance it covered or the final position, their judgments tend to align with the visual effect rather than the felt actual movement. Crucially, however, when incongruency becomes too large, this bias has been shown to disappear, and participants regain their ability to judge their movement quite accurately (Ladwig et al. 2012).

Knoblich and Kircher (2004) not only varied the extent of visuo-motor incongruency, but also measured participants’ awareness of it. In their paradigm, participants produced continuous circular movements of a target on a screen by moving a pen in the same trajectory on a graphics tablet. Initially, the cursor mirrored the pen perfectly. At some point, however, a gain of varying size was introduced to the cursor movement, requiring participants to reduce the radius of their pen movements to maintain the same visual trajectory. Strikingly, individuals were not only faster and more likely to detect higher gains, but they would also adjust their movements successfully even when failing to detect the gain in the first place. Such implicit and explicit adaptation to visuomotor conflict have been shown to differ in terms of temporality, dominance, specificity, or stability (e.g., (Bond and Taylor 2015; Hegele and Heuer 2010; Mazzoni and Krakauer 2006; Neville and Cressman 2018; Taylor et al. 2014; Werner et al. 2015)). However, the underlying changes in the way the cognitive system produces those adaptations have been less well investigated.

The present study investigated how introducing visuo-motor incongruency—and, critically, individuals’ awareness of it—affects the up- and downregulation of body-related effect codes during action. As a proxy for this regulation process, we measured tactile sensitivity at the moving effector (Kloss and Kunde 2026), [31]. An extensive body of research suggests that tactile events are attenuated during movement, a phenomenon known as tactile suppression (for a review, see (Juravle et al. 2017)). Strinkingly, this is already the case before movement onset, suggesting that the representation of body- and environment-related effects at the stage of movement planning modulates tactile sensivitiy (e.g., (Colino and Binsted 2016; Voss et al. 2008)). When participants have reason to attend to haptic action effects, however, they appear to become more responsive to tactile stimuli (Juravle and Deubel 2009; Juravle et al. 2013; Voudouris and Fiehler 2017, 2022). Therefore, tactile sensivity offers a direct means to track the up- and downregulation of haptic action effects we are expecting.

Subjects performed continuous circular movements that were transformed into either perfectly congruent or more or less incongruent movements of a visual target (Knoblich and Kircher 2004). Visuo-motor incongruency was introduced a few seconds before the tactile probe, and participants could indicate whether they had noticed a change in the relationship between their movement and the visual effects without stopping their movement. This design allowed us to compare tactile sensitivity not only between congruent and incongruent transformations but also between incongruent transformations that were consciously detected versus those that were not—while controlling for the degree of incongruency.

Experiment 1 aimed to replicate the findings by Knoblich and Kircher (2004) and extend them by probing tactile sensitivity as a function of visuo-motor incongruency and agents’ awareness to assess the dynamic up- and downregulation of body-related effect codes. Experiment 2 introduced only a small, but significant design change that increased participants’ need to compensate for incongruency. Methods are reported jointly, with differences between experiments highlighted where relevant.

## Methods

A power analysis in R version 2025.05.1 indicated that 66 participants would yield a minimum power of 1-β = 0.8 to detect a small-to-medium effect size (*d*_*z*_ = 0.35) at α = 0.05 across the two studies. When it became clear that recruiting 66 eligible participants for each experiment was not feasible, we added a note to our preregistration specifying that data collection would stop on 11/16/2025. Figure 5 in the Appendix illustrates the development of *p*-values and effect sizes for the main analysis throughout data collection, illustrating that we did not terminate recruitment at an opportunistic moment.

Data collection was stopped after 91 participants for Exp. 1 and 75 participants for Exp. 2, recruited via Sona Systems (https://www.sona-systems.com/), had provided informed consent to participate. Exclusion rates were fairly high because our design involved two variables strongly influenced by inter- and intraindividual variability: sensitivity to detect subtle visuo-motor incongruencies and sensitivity to detect weak tactile stimuli. After applying the exclusion criteria detailed in the Results section, 49 eligible datasets (*M*_age_ = 26 years, 40 female, 1 diverse) were included in the main analyses of Experiment 1, and 34 datasets (*M*_age_ = 27 years, 27 female) in Experiment 2.

## Materials and apparatus

**Fig. 1:**
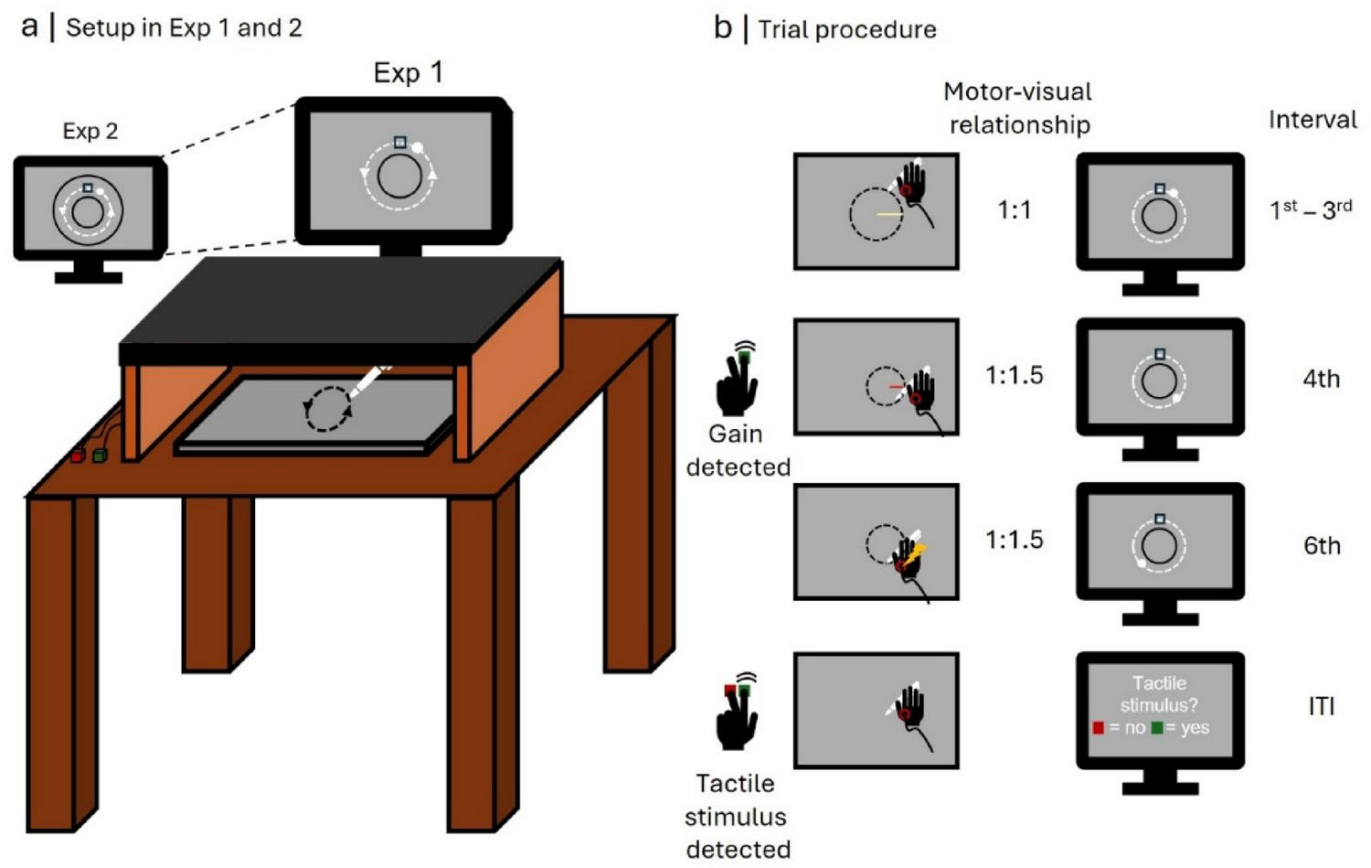
Laboratory setup (**a**) and visualization of an incompatible trial with tactile stimulation (**b**). *Note*: The trial depicted in (**b**) visualizes a perfect adaptation where the subject compensates the gain by decreasing their movement radius (yellow vs. red radius) to the extent needed to produce the exact same visual effect as before the introduction of incongruency. Participants could indicate they had detected a gain by pressing a key while continuing to perform their movements. The yellow lightning bolt represents the tactile stimulation on the effector within the sixth interval

Figure 1 illustrates the basic setup used in Experiments 1 and 2. Participants were seated approximately 70 cm from a 24-inch screen. Using a digital pen, they performed circular movements with their right hand on a graphics tablet (Intuos 4 XL, Wacom Co., Ltd., Krefeld, Germany) that were translated into corresponding circular movements of a white dot displayed on the screen. A wooden box and a black cloak draped around participants’ necks prevented them from seeing their hand movements.

At the center of the screen, a black circle with a radius of 3.5° visual angle was presented, along with a small black box positioned 1.5° above it. In Experiment 2, this display was extended by a second, larger black circle with a radius of 6.5°. Two response keys were placed to the left of the box, allowing participants to respond using the index and middle fingers of their left hand.

Additionally, we attached a vibro-tactile stimulation device (Dancer Design, Ingleton, United Kingdom) to the skin between the thumb and index finger of their right hand and handed participants a pair of headphones.

### Procedure

In the first of two practice blocks (40 trials), participants familiarized themselves with the basic task. To start a trial, they moved the white dot on the screen into the small box using the pen. Once positioned, an auditory timer played short, consecutive tones every two seconds through the headphones. Simultaneously, a green dot appeared and began moving counterclockwise along a circular trajectory with a radius of 5° around the screen center at a speed of two seconds per interval, reaching the 12 o’clock position in sync with each tone. Participants were instructed to closely follow the green dot with the white dot by reproducing the same circular trajectory on the tablet. Pen movements on the tablet were transformed in a 1:1 manner into dot movements on the screen.

This block also served to determine participants’ tactile detection thresholds using an adaptive up-down staircase procedure (e.g., (Leek 2001)). At a randomly varying moment in between 500 and 1000 ms after initiating the sixth and final interval, participants received a weak tactile stimulus on their moving hand. After completing the interval, they reported whether they had perceived the stimulus using the two response keys. If they detected it, the next trial’s stimulus intensity was increased by one unit; if they had not, it was decreased by one unit. Every ten trials, those units were halved to refine the threshold estimate. The first trial used a displacement of the vibrating element of 21 µm, with the initial (largest) adjustment step of 2 µm. The element was displaced by 41 µm at the largest and by 1 µm at the smallest intensity level. After 40 trials, we computed the average intensity of the last ten reversals (i.e., trials where detection status switched relative to the previous trial). Based on this average reversal intensity, we selected one out of 321 possible stimulus intensities for the experiment.

Next, participants completed twelve trials in a second practice block. They were instructed to move the white dot as before, performing one interval every two seconds. However, we did not present the green dot any longer. Instead, they had to control their movement speed solely by matching the rhythm of the tones, reaching the 12 o’clock position whenever a tone was played.

In this block, participants were introduced to the manipulation of visuo-motor (in-)congruency. In 50% of trials, at a randomly varying moment within the fourth interval, the 1:1 transformation of pen movement into dot movement was replaced by a 2:1 transformation by introducing a 100% gain to the dot movement. This caused the dot to move two units in one direction when the pen was moved one unit in that same direction. Participants were told to expect such a change in the relationship between pen and dot movements in some trials and press the left of the two response keys as soon as they detected it. They were also instructed to continue moving the dot along the same circular trajectory, which required reducing the radius of their pen movements after the gain was introduced.

In 50% of trials, participants received a tactile stimulus during the sixth interval, with the intensity set to their individual detection threshold. After completing this final interval, they judged whether a stimulus had been presented.

Finally, participants performed the main task, which differed from the second practice block only in the range of visuo-motor transformation levels: Instead of a single 100% gain, we introduced a set of varying gains: 0% (no gain), 20%, 27.5%, 35%, 42.5%, 50%, 57.5%, 65%, and 72.5%. We expected that at the extreme ends of this gain continuum, the change in the visuo-motor relationship would be detected almost never (for low gains) or almost always (for high gains). Since we aimed to compare tactile sensitivity in trials where visuo-motor incongruency was detected vs. not detected, those gain levels (20, 27.5, 65, and 72.5%) were presented only in ten trials each. The remaining gain levels (0, 35, 42.5, 50, 57.5%) were probed in 40 trials each. At each level, half of all trials included a tactile stimulation within the sixth interval. Figure 1.b illustrates an incongruent trial with tactile stimulation.

This setup allowed us to study tactile sensitivity not only under visuo-motor congruency (i.e., at a gain level of 0%) and varying levels of incongruency (i.e., at gain levels > 0%), but also as a function of incongruency detection.

## Results

From all analyses, we excluded trials when i) two or more interval durations deviated more than 500 ms from the duration of 2000 ms instructed by the tone rhythm (Exp. 1: on average, 9% of all trials per subject; Exp. 2: 11%), and ii) participants indicated having detected incongruency before it was applied (Exp. 1: 4%; Exp. 2: 4%). In Exp. 2, we further excluded all trials where the white dot left the track iii) three or more times or for more than one second at a time (12%). From tactile sensitivity analyses, we further excluded trials when iv) the tactile stimulus was applied after the end of the sixth interval (Exp. 1: 17% of all tactile stimulation trials; Exp. 2: 24%), v) incongruency was falsely detected (Exp. 1: 7% of all congruent trials; Exp. 2: 6%), and vi) trials where participants indicated having detected incongruency after the tactile stimulus was applied (Exp. 1: 4% of all tactile stimulation trials; Exp. 2: 3%).

Next, we excluded vii) subjects with tactile detection rates < 15% or > 85% (Exp. 1: 31 subjects; Exp. 2: 33 subjects) as well as viii) subjects who had fewer than 5 observations in the relevant cells for the tactile sensitivity analysis (Exp. 1: 7 subjects; Exp. 2: 3 subjects). Finally, we excluded subjects when ix) incongruency detection rates were clearly unrelated to the extent of incongruency (Exp. 1: 1 subject; Exp. 2: 0 subjects), and x) when technical equipment had failed during the experiment (Exp. 1: 3 subjects; Exp. 2: 1 subject).

Note that many of these trial-level exclusions overlapped and that those subjects with many exclusions were also most likely to be excluded on the subject-level. For the remaining subjects, an average of 7% of all trials (15% in Exp. 2) were excluded from all analyses and an additional 6% (4% in Exp. 2) from all tactile sensitivity analyses.

As already described in the Methods section, we faced a particular challenge in all analyses where we compared trials in which the visual gain was detected vs. not detected or focused solely on trials in which it was not: For the extreme gain levels (i.e., at very low or high gains), either of the two cells had only very few observations (if any) for each subject. To account for this, all those analyses included only trials with the gain levels 0%, 35%, 42.5%, 50%, and 57.5%.

Except for the analysis of *d*′, which requires collapsing data across several trials, all hypothesis s were conducted using trial-level linear mixed-effects models. As preregistered, each model included subject-specific random intercepts and, following the maximal random-effects principle (Barr 2013), random slopes for each fixed effect. When singular fits or convergence issues occurred, we reduced complexity by removing random slopes—starting with interaction terms, then main effects with the smallest variance contribution. Fixed effects were evaluated using likelihood ratio tests [45]. Note that we did not proceed in the planned stepwise manner we had preregistered but evaluated the significance of each effect by testing if the full model explained significantly more variance than a model without the effect (and corresponding random slopes) to ensure adequate hypothesis testing. When the full model included an interaction, we assessed the significance of main effects by comparing a model without interaction and a model without interaction and without the respective effect. Additionally, we decided to center the size of the visual gain on subject level.

### Experiment 1

**Fig. 2:**
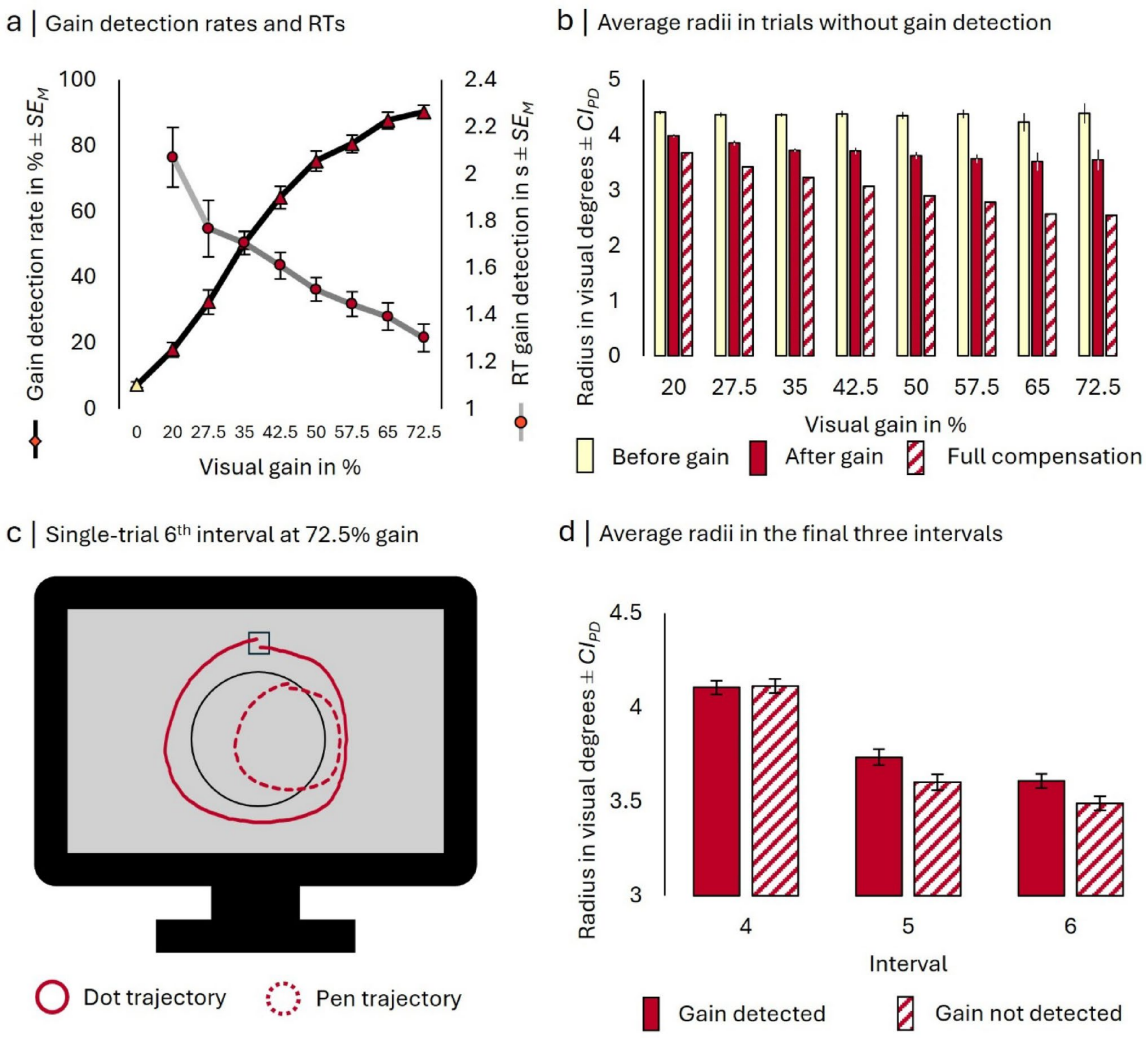
Gain detection performance and movement characteristics. *Note*: **a** Mean gain detection rates (black line, left y-axis) and response times (gray line, right y-axis) as a function of gain size. **b** Average radii in trials where the gain was not detected before it was introduced and after it was introduced. The patterned bars represent the hypothetical radii necessary to achieve the exact same dot radius as before the introduction of the visual gain (i.e., perfect compensation). Error bars represent 95% confidence intervals of paired differences (Pfister and Janczyk 2013) for the comparison of radii before and after the gain was introduced at each gain size. **c** Pen trajectory and its resulting dot trajectory from a single trial’s 6th interval. The participant compensated very well for the gain of 72.5%, producing more or less an ideal visual effect. In this trial, the gain had been introduced when the participant had been moving along the 3 o’clock position within the 4th interval. **d** Average radii in the final three intervals of trials where a visual gain was introduced within the fourth interval as a function of gain detection. Error bars represent confidence intervals of paired differences (Pfister and Janczyk 2013) for the comparison of radii in trials where the gain was detected vs. not detected

**Fig. 3:**
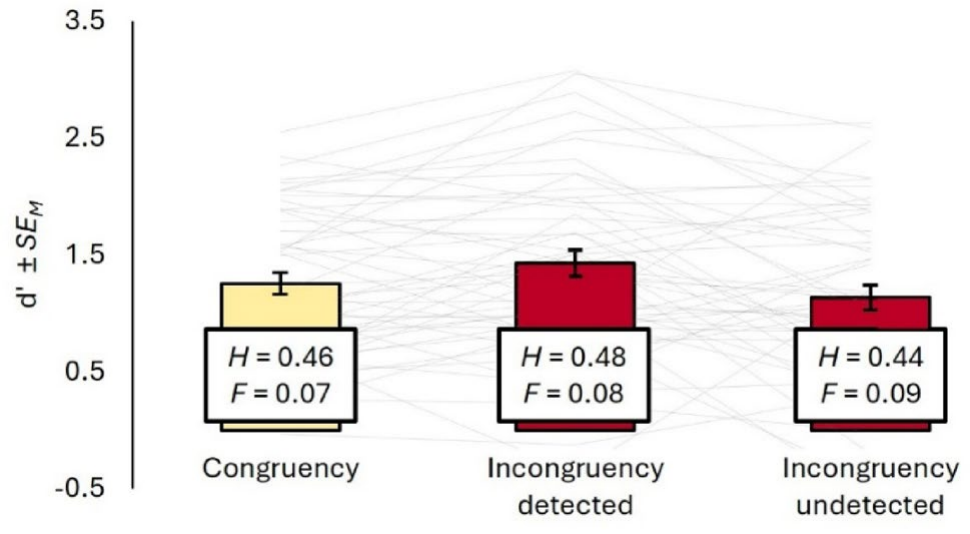
Tactile sensitivity as a function of visuo-motor (in-)congruency and its detection. *Note*: Tactile sensitivity *d’* as well as the underlying Hit (*H*) and False alarm (*F*) rates for congruent trials and trials where incongruency was detected vs. not detected. Congruent trials had a gain level of 0%, while incongruent trials contained the gain levels 35, 42.5, 50, and 57.5%. Gray lines represent individual means. Error bars represent standard errors of the mean (Pfister and Janczyk 2013)

The replications of the findings on visuo-motor adaptation and gain detection by Knoblich and Kircher (2004) are visualized in Fig. 2, while Fig. 3 summarizes our novel tactile sensitivity results.

### Gain detection, compensation, and aftereffects

To test if participants did in fact detect higher gains more easily, we fitted a logistic linear mixed-effects model predicting gain detection from gain size, including random slopes for the effect of gain size by subject. Gain size significantly influenced detection, χ^2^(3) = 4628.9, *p* < 0.001, *AIC*_*reduced*_ = 14,386.6, *AIC*_*full*_ = 9763.7. The odds to detect a gain increased with its size, *OR* = 6.58, 95% *CI* [5.41, 8.01], *z* = 18.87, *p* < 0.001.

Additionally, we ran a linear mixed-effects model on the response times of gain detection including a fixed effect and random slopes for the size of the gain. Accounting for gain size significantly improved fit, χ^2^(3) = 125.74, *p* < 0.001, *AIC*_*reduced*_ = 8502.1, *AIC*_*full*_ = 8382.4. Larger gains were detected more quickly, *β* = -0.08, 95% *CI* [-0.11, -0.06], *t* = -7.18.

Next, we scrutinized whether participants compensated for the introduction of a visual gain even when they failed to detect it. We computed a linear mixed-effects model on the average radius across the entire trial with the fixed effects gain size, gain introduction, and their interaction. Again, accounting for whether the gain had been introduced yet significantly improved model fit, χ^2^(3) = 4022.7, *p* < 0.001, *AIC*_*reduced*_ = 6060.8, *AIC*_*full excl interaction*_ = 2044.1. Gain size also explained significant variance, χ^2^(1) = 39.52, *p* < 0.001, AIC_*reduced*_ = 2081.6, as did the interaction, χ^2^(1) = 41.74, *p* < 0.001, AIC_*reduced*_ = 2044.1, AIC_*full*_ = 2004.4. Once the gain had been introduced, the average radius decreased with gain size, *β* = -0.06, 95% *CI* [-0.07, -0.04], *t* = -9.05, p < 0.001. At a constant gain, the radius was larger before than after its introduction, *β* = 0.68, 95% *CI* [0.64, 0.72], *t* = 33.33, p < 0.001, and the effect of gain size was (unsurprisingly) larger once it had been introduced, *β* = 0.06, 95% *CI* [0.04, 0.07], *t* = 6.47, p < 0.001. After their introduction, larger gains were compensated with smaller pen movements. We also replicated the analysis of the radius at the 12 o’clock position of the third and fourth interval (Knoblich and Kircher 2004) and report it in the Appendix.

To confirm that participants did adjust their movement in response to all four gain levels that would go into the analysis of tactile sensitivity, we performed separate within-subjects *t*-tests for each level, comparing the average radius before vs. after the gain was introduced in trials where it was not detected (all *p*-values are Bonferroni adjusted based on the four tests computed). As expected, average radii decreased after a 35% gain was introduced, *t*(48) = 33.35, *p* < 0.001, *d*_*z*_ = 4.76, and the same held for the 42.5% gain, *t*(47) = 23.07, *p* < 0.001, *d*_*z*_ = 3.33, the 50% gain, *t*(44) = 23.64, *p* < 0.001, *d*_*z*_ = 3.52, and the 57.5% gain, *t*(41) = 20.38, *p* < 0.001, *d*_*z*_ = 3.14.

Next, we computed exploratory paired t-tests analyzing the average radius in incongruent trials as a function of interval and gain detection. The average radius in the fourth interval (where the gain was introduced at some point) was not yet affected by gain detection, *t*(48) = 0.35, *p* = 0.727, *d*_*z*_ = 0.05. In the fifth interval, however, it was smaller in trials where the gain was detected, *t*(48) = 6.37, *p* < 0.001, *d*_*z*_ = 0.91, and the same was the case in the sixth interval, *t*(48) = 6.41, *p* < 0.001, *d*_*z*_ = 0.92.

As another indicator of gain adaptation, we explored the aftereffects of having experienced a gain in the previous trial (note that we had not preregistered this analysis). Specifically, we modeled the average radius in the first interval of trial *n* as a function of gain size and detection in trial *n*—1. The full model included the fixed effects gain size and gain detection in trial *n*—1, their interaction, and random slopes for the effects of gain detection. Accounting for the size of the gain in trial *n*—1 significantly improved model fit, χ^2^(1) = 483.95, *p* < 0.001, *AIC*_*reduced*_ = 2542.0, *AIC*_*full excl interaction*_ = 2060.1. When the gain was not detected in trial *n*—1, the average radius in trial *n* decreased with higher gains in trial *n*—1, *β* = -0.09, 95% *CI* [-0.10, -0.08], *t* = -24.00. Including gain detection also improved model fit, χ^2^(3) = 39.26, *p* < 0.001, *AIC*_*reduced*_ = 2093.3. However, at an average gain size, having detected the gain only descriptively increased the average radius, *β* = 0.02, 95% *CI* [-0.01, 0.04], *t* = 1.38, suggesting an interaction of gain size and detection. Indeed, accounting for this interaction further improved model fit, χ^2^(1) = 12.49, *p* < 0.001, *AIC*_*reduced*_ = 2060.1, *AIC*_*full*_ = 2049.6. The effect of gain size was larger when the gain was detected, *β* = 0.03, 95% *CI* [0.01, 0.05], *t* = 3.53. Thus, participants adapted more to larger gains than smaller gains, which were detected rather than undetected.

Finally, we explored how participants’ ability to maintain circular movements was affected by gain introduction and its detection. Since such performance parameters were not the central focus of our study, we report these results in the Appendix (see also Fig 6).

### Tactile sensitivity

As a proxy for the up- or downregulation of body-related effect codes, we measured the sensitivity to tactile events on the moving effector within the final interval of a trial. To analyze tactile sensitivity as a function of visuo-motor incongruency and agents’ awareness of it, we grouped all trials i) without visual gain where no gain was detected (i.e., visuo-motor congruency), ii) with visual gain between 35% and 57.5% where the gain was detected (i.e., visuo-motor incongruency detected), and iii) with visual gain between 35% and 57.5% where the gain was not detected (i.e., visuo-motor incongruency undetected). Note that this does not constitute a 2 (Congruency vs. Incongruency) × 2 (Incongruency detected vs. not detected) design, as trials without visual gain but with reported detection were (predictably) very rare for most participants. All tactile sensitivity results are summarized in Fig. 3.

**Fig. 4:**
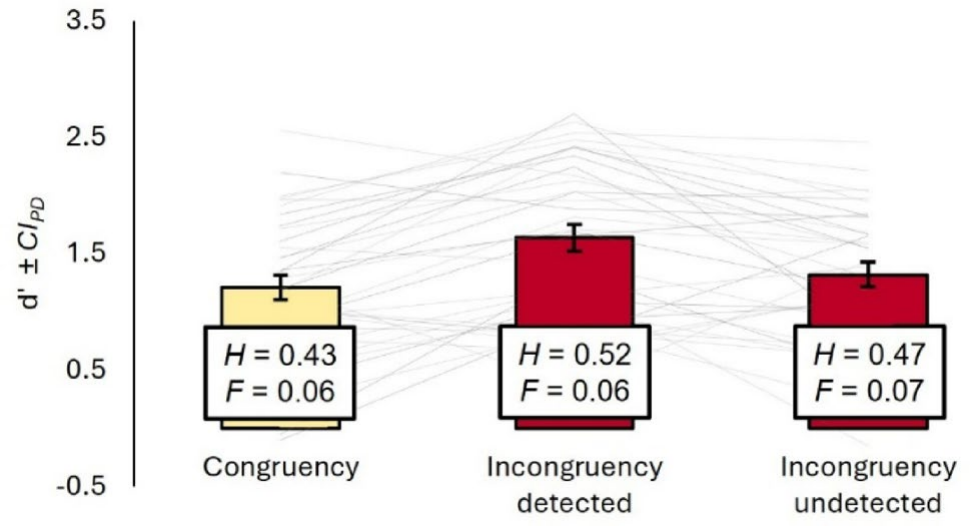
Tactile sensitivity as a function of visuo-motor (in-)congruency and its detection. *Note*: Tactile sensitivity d’ as well as the underlying Hit (*H*) and False alarm (*F*) rates for congruent trials and trials where incongruency was detected vs. not detected. Congruent trials had a gain level of 0%, while incongruent trials contained the gain levels 35, 42.5, 50, and 57.5%. Error bars represent standard errors of the mean (Pfister and Janczyk 2013)

**Fig. 5:**
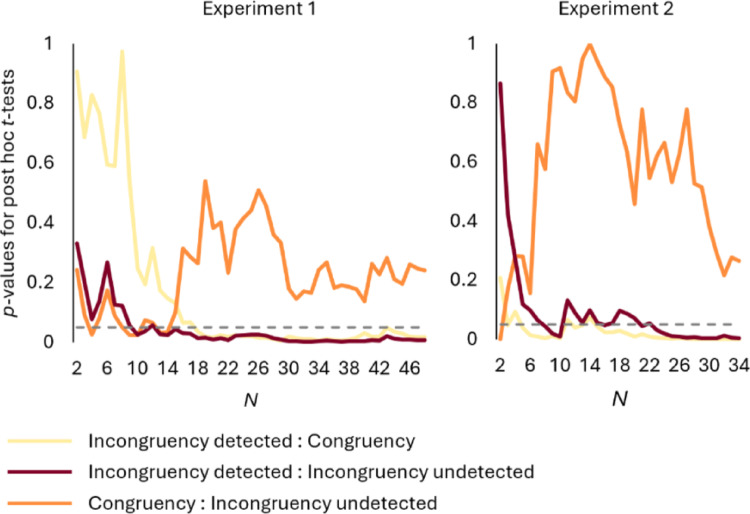
Development of p-values for the post hoc t-tests on d’ in Exp. 1 and 2

**Fig. 6:**
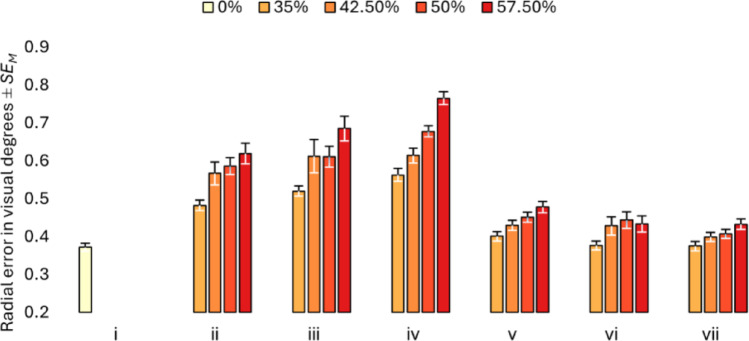
The average radial error in Exp. 1 as a function of gain size and detection within different intervals of interest. *Note*: The average radial error (i) before a gain was introduced, (ii) after a gain was introduced in trials where it was not detected, (iii), after a gain was introduced in trials where it was not detected, limited to an interval corresponding to the average detection time in trials where it was detected, (iv) after a gain was introduced but before it was detected, (v) after a gain was detected, (vi) in the last interval of trials where a gain was not detected, and (vii) in the last interval of trials where incongruency was detected

Within each group, we computed the sensitivity measure *d*’ and the response criterion *c* from the signal detection theory framework (Macmillan et al. 2001) using the “psycho” package in R. We identified Hits as trials where a participant reported having detected a tactile stimulus when one was present, Misses as trials where they reported they had not detected a stimulus when one was present, False Alarms as trials where they reported having detected a stimulus when none was present, and Correct Rejections as trials where a participant reported they had not detected a stimulus when none was present. When any cell contained zero observations, we applied a correction by adding 0.5 and subtracting 0.5 from the corresponding paired cell (e.g., when a participant had zero False Alarms in one condition, we added 0.5 False Alarms while subtracting 0.5 Correct Rejections; (Kadlec 1999)) (Fig. 4).

We entered *d*’ into a repeated-measures Analysis of Variance (ANOVA) with one factor (Congruency vs. Incongruency detected vs. Incongruency undetected) and observed a main effect, *F*(2, 96) = 5.16, *p* = 0.007, η_p_^2^ = 0.10. Post hoc *t*-tests revealed that tactile sensitivity was significantly higher in trials with detected incongruency (*M* = 1.43, *SD* = 0.79) compared to trials with undetected incongruency (*M* = 1.14, *SD* = 0.74), *t*(48) = 2.81, *p* = 0.007, *d*_*z*_ = 0.40, and congruent trials (*M* = 1.25, *SD* = 0.64), *t*(48) = 2.25, *p* = 0.029, *d*_*z*_ = 0.32. However, sensitivity in undetected incongruency trials did not differ significantly from congruent trials, *t*(48) = 1.30, *p* = 0.198, *d*_*z*_ = 0.19.

The response criterion *c* was analyzed in the same manner. The ANOVA revealed no main effect, *F*(2, 96) = 0.56, *p* = 0.572, η_p_^2^ = 0.01, and none of the post hoc *t*-tests were significant (all *p* > 0.366).

Since larger visual gains were detected more frequently, trials with larger gains were more likely to be sorted into the group of trials with detected than undetected incongruency. It appears unlikely that the differences between sensitivity in incongruent trials are driven by this gain size confound, given that tactile sensitivity was not further reduced in congruent trials where the gain size was zero. Still, we had Preregistered to test if the effect of incongruency detection would persist when controlling for gain size. Since the two predictors gain size and gain detection were, however, strongly correlated (variance inflation factor, *VIF* = 5.67) and thus inconclusive, the results of this analysis will only be reported in the Appendix. Instead, we computed two separate linear regressions testing the effect of average gain size on d’ for each of the detection conditions. Gain size did not predict tactile sensitivity when incongruency was detected, *R*^2^ = 0.01, β = 0.07, *p* = 0.431, nor when it was not detected, *R*^2^ < 0.01, β = 0.02, *p* = 0.687. As another approximation of how much gain size affected tactile sensitivity, we computed a trial-level generalized mixed model on the accuracy of tactile responses in trials with a visual gain. Gain detection itself did however not significantly improve fit over a random intercept only model, χ^2^(1) = 1.39, *p* = 0.238, *AIC*_*reduced*_ = 8359.4, *AIC*_*full*_ = 8360.5, with the odds to respond correctly increasing only descriptively when a gain was detected, *OR* = 1.08, 95% *CI* [0.95, 1.22], *z* = 1.18, *p* = 0.236. Additionally controlling for gain also not improve model fit either, χ^2^(1) = 0.20, *p* = 0.203, *AIC*_*full*_ = 8361.8, and the odds to respond correctly remained virtually the same, *OR* = 1.07, 95% *CI* [0.94, 1.22], *z* = 1.02, *p* = 0.308.

Finally, we explored if differences in tactile sensitivity might be driven by the speed of the moving effector (Colino and Binstead 2016; (Cybulska-Klosowicz et al. 2011)). We computed the average velocity of the pen in trials where a tactile stimulus was applied in an interval from 100 ms before to 100 ms after the onset of the movement. In Exp. 1, the pen was moved considerably faster in congruent trials (*M* = 15.18 a.u.) compared to trials where incongruency was detected (*M* = 12.33 a.u.), *t*(48) = 16.7, *p* < 0.001, *d*_*z*_ = 0.76, and trials where incongruency was missed (*M* = 12.75 a.u.), *t*(48) = 11.5, *p* < 0.001, *d*_*z*_ = 0.62. There was also a small difference between the latter two, *t*(48) = 2.21, *p* = 0.032, *d*_*z*_ = 0.38. This pattern is not surprising, considering that participants responded to visual gains by drawing smaller circles in the same amount of time, and they did so to a slightly greater extent after having detected the gain. To better assess if the speed itself had an influence on tactile stimulus detection, we computed separate generalized linear mixed effects models for each condition. The average velocity in the 200 ms interval around the onset of tactile stimuli did not predict their detection in congruent trials, χ^2^(1) = 0.32, *p* = 0.569, *AIC*_*reduced*_ = 1132.8, *AIC*_*full*_ = 1134.5, nor in trials where incongruency was detected, χ^2^(1) = 0.86, *p* = 0.353, *AIC*_*reduced*_ = 3010.7, *AIC*_*full*_ = 3011.8. It did however in trials where incongruency was not detected, χ^2^(1) = 5.67, *p* = 0.017, *AIC*_*reduced*_ = 1414.8, *AIC*_*full*_ = 1411.2.

In an exploratory analysis, we examined whether increased tactile sensitivity might precede, rather than follow, the detection of visuo-motor incongruency. Specifically, we analyzed the sensitivity to visuo-motor incongruency as a function of the detection of a tactile stimulus in the previous trial. Notably, the temporal interval between gain introduction in trial n and the tactile stimulus in trial n was comparable to the interval between the tactile stimulus in trial n–1 and gain introduction in trial n. Thus, we compared sensitivity to visuo-motor incongruency in trials where the preceding tactile stimulus was detected versus not detected. 

Hits were defined as trials where participants reported detecting a gain when one was present; Misses as trials where they did not detect a gain when one was present; False Alarms as trials where they reported detecting a gain when none was present; and Correct Rejections as trials where they correctly reported no gain when none was present. We entered the corresponding *d’* values into a within-subjects *t*-test. There was no difference in sensitivity to the visual gain when the preceding tactile stimulus was detected compared to when it was not, *t*(47) = 0.15, *p* = 0.879, *d*_*z*_ = 0.02. This lends some support to the idea that gain detection increased tactile sensitivity, rather than being the consequence of temporarily increased tactile sensitivity. These results should be treated with caution, however, as the number of noise trials without gain was very limited overall and relative to signal trials with gain (~ 20% no gain, 80% gain).

### Brief discussion

Experiment 1 replicated key observations of the study by Knoblich and Kircher (2004). Participants’ detection of visuo-motor incongruencies increased with the size of such incongruencies, while motoric adaptation occurred even when incongruency went unnoticed. More importantly, we observed that sensitivity to tactile stimulation at the moving hand was larger in trials where visuo-motor incongruencies were noted compared to when they were not. This suggests that awareness of such incongruencies upregulates the processing of body-related action feedback. The alternative model that increased processing of body-related feedback and sensitivity to visuo-motor incongruencies just co-occur was not supported as tactile detection performance in immediately preceding trials did not predict whether a visuo-motor incongruency in the subsequent trial would be noted or not.

The visual trajectories of the cursor in Experiment 1 were not very much constrained, and participants were not forced to compensate for the introduction of a visual gain. While they still did so to some extent, Experiment 2 was designed to ensure that visual gains caused substantial changes in bodily movements that participants could or could not detect.

### Experiment 2

The design of the second study closely mirrored that of the first, with one key difference: participants were required to keep the dot within predefined screen boundaries, thereby increasing the need to compensate for visual gain. To verify the effectiveness of this manipulation, we computed the mean difference in radii before and after the introduction of a gain for each gain size and each subject, then averaged the differences from each gain to obtain a single adaptation score per subject. We compared these scores between Experiment 1 and 2 in a between-subjects *t*-test. As intended, participants reduced the size of their movements in response to the gain to a greater extent in Experiment 2, *t*(79.9) = 5.65, *p* < 0.001, *d* = 1.23.

### Gain detection, compensation, and aftereffects

Again, we tested whether gain detection was influenced by gain size. Including gain size and random slopes significantly improved model fit compared to a random-intercepts-only model, χ^2^(3) = 3037, *p* < 0.001, *AIC*_*reduced*_ = 8912.6, *AIC*_*full*_ = 5881.6. The odds of detecting the gain increased with its size, *OR* = 7.43, 95% *CI* [5.79, 9.53], *z* = 15.77, p < 0.001.

As for Exp. 1, we ran a linear mixed-effects model on the response times of gain detection. Gain size significantly affected detection time, χ^2^(1) = 95.37, *p* < 0.001, *AIC*_*reduced*_ = 4231.4, *AIC*_*full*_ = 4138.0, with larger gains detected more quickly, *β* = -0.09, 95% *CI* [-0.11, -0.07], *t* = -9.85.

The analysis of average radii revealed the same results pattern as in the first experiment. The full model included fixed effects for gain size, gain introduction, their interaction, and random slopes for the effect of gain introduction. Accounting for the size of the gain improved model fit, χ^2^(1) = 142.93, *p* < 0.001, *AIC*_*reduced*_ = -298.5, *AIC*_*full excl interaction*_ = -439.38, and so did adding whether this gain had been introduced yet, χ^2^(3) = 4643.7, *p* < 0.001, *AIC*_*reduced*_ = -439.4. The interaction also explained significant variance, χ^2^(1) = 91.68, *p* < 0.001, *AIC*_*reduced*_ = -439.4, *AIC*_*full*_ = -529.1. When the gain had already been introduced, the average radius decreased with higher visual gains, *β* = -0.09, 95% *CI* [-0.10, -0.07], *t* = -15.50, p < 0.001. At constant gain size, it was (unsurprisingly) larger before the gain change, *β* = 0.83, 95% *CI* [0.79, 0.86], *t* = 50.39. So, even when incongruency remained undetected, participants compensated for larger gains with smaller pen movements.

Again, we performed separate within-subjects *t*-tests on each gain level that would go into the analysis of tactile sensitivity comparing the average radius before vs. after the gain was introduced in trials where it was not detected (all *p*-values are Bonferroni adjusted). Once more, average radii decreased after a 35% gain was introduced, *t*(32) = 51.01, *p* < 0.001, *d*_*z*_ = 8.88, and they did so for the 42.5% gain, *t*(32) = 25.80, *p* < 0.001, *d*_*z*_ = 4.49, the 50% gain, *t*(29) = 25.85, *p* < 0.001, *d*_*z*_ = 4.72, and the 57.5% gain, *t*(28) = 16.45, *p* < 0.001, *d*_*z*_ = 3.05.

Exploratory paired *t*-tests analyzing the average radius in incongruent trials as a function of interval and gain detection revealed the same pattern as in Exp. 1. The average radius in the fourth interval (where the gain was introduced at some point) was not affected by gain detection, *t*(32) = 0.95, *p* = 0.350, *d*_*z*_ = 0.17. In the fifth interval, however, it was smaller in trials where the gain was detected, *t*(32) = 6.97, *p* < 0.001, *d*_*z*_ = 1.21, and the same was the case in the sixth interval, *t*(32) = 6.67, *p* < 0.001, *d*_*z*_ = 1.16.

As before, we observed substantial aftereffects of gain adaptation. The full model included fixed effects for gain size and gain detection in trial *n*—1, their interaction, and random slopes for gain detection. Gain size in trial *n*—1 significantly affected the average radius in the first interval of trial *n*, χ^2^(1) = 369.34, *p* < 0.001, *AIC*_*reduced*_ = -979.97, *AIC*_*full excl interaction*_ = -1347.3. Detection of gain in trial *n*—1 did not explain additional variance, χ^2^(3) = 6.12, *p* = 0.106, *AIC*_*reduced*_ = -1347.2. There was however a significant interaction between gain size and detection, χ^2^(1) = 23.49, *p* < 0.001, *AIC*_*reduced*_ = -1347.3, *AIC*_*full*_ = -1368.8. The average radius in trial *n* decreased with higher gains in trial *n*—1, *β* = -0.09, 95% *CI* [-0.10, -0.08], *t* = -19.13, p < 0.001, and this effect was larger when the gain was detected, *β* = 0.04, 95% *CI* [0.03, 0.06], *t* = 4.86, p < 0.001.

Analyses of the average radius across the entire trial and of radial errors closely replicated the results of Exp. 1 and are reported in the Appendix.

### Tactile sensitivity

We analyzed d’ as in Exp. 1 and again observed a main effect of condition (congruent vs. incongruent-detected vs. incongruent-undetected), *F*(2, 66) = 10.46, *p* < 0.001, η_p_^2^ = 0.24. Post hoc *t*-tests revealed the same pattern as before: tactile sensitivity was significantly higher in trials with detected incongruency (*M* = 1.64, *SD* = 0.68) compared to trials with undetected incongruency (*M* = 1.32, *SD* = 0.60), *t*(33) = 3.09, *p* = 0.004, *d*_*z*_ = 0.53, and congruent trials (*M* = 1.21, *SD* = 0.63), *t*(33) = 4.70, *p* < 0.001, *d*_*z*_ = 0.81. However, sensitivity in undetected incongruency trials did not differ significantly from congruent trials, *t*(33) = 1.13, *p* = 0.266, *d*_*z*_ = 0.19. The corresponding ANOVA of the response criterion c revealed no main effect, *F*(2, 66) = 0.90, *p* = 0.410, η_p_^2^ = 0.03, and none of the post hoc *t*-tests were significant (all *p* > 0.061).

Again, we tested if gain size affected tactile sensitivity. Once more, a linear mixed model with the effects gain size and gain detection suffered from multicollinearity (*VIF* = 6.64), so we computed two separate linear regressions for both detection conditions. Gain size did not predict tactile sensitivity when incongruency was not detected, *R*^2^ = 0.03, *β* = -0.05, *p* = 0.307. When it was detected, however, smaller average gains were associated with larger d’ values, *R*^2^ = 0.14, *β* = -0.15, *p* = 0.028. This suggests that the positive effect of incongruency detection was not driven by gain size – if anything, it was restrained by it. This was further supported by a trial-level generalized mixed model on the accuracy of tactile responses in trials with a visual gain. Gain detection significantly improved fit over a random intercept only model, χ^2^(1) = 5.60, *p* = 0.018, *AIC*_*reduced*_ = 5142.0, *AIC*_*full*_ = 5138.4, with the odds to respond correctly increasing when a gain was detected, *OR* = 1.20, 95% *CI* [1.03, 1.40], *z* = 2.38, *p* = 0.017. Controlling for gain did not further improve model fit, χ^2^(1) = 1.90, *p* = 0.169, *AIC*_*full*_ = 5138.5, and the odds to respond correctly when the gain was detected even increased slightly, *OR* = 1.25, 95% *CI* [1.06, 1.47], *z* = 2.70, *p* = 0.007.

Finally, we analyzed the sensitivity to visuo-motor incongruency as a function of the detection of a tactile stimulus in the previous trial. Again, there was no difference in sensitivity to the visual gain when the preceding tactile stimulus was detected compared to when it was not, *t*(32) = 0.63, *p* = 0.535, *d*_*z*_ = 0.11.

## Discussion

In two experiments, we investigated how visuo-motor incongruency and agents’ awareness of it influence the representation of body-related effect codes during action. Participants produced continuous circular movements with a pen that controlled a dot on a screen. In some trials, a gain of varying size was introduced to the transformation, requiring participants to adjust their movement to maintain the same visual trajectory (Knoblich and Kircher 2004). Tactile sensitivity toward the end of each trial served as a proxy for the up- and downregulation of body-related effect codes.

A large body of research suggests that bodily effects are typically neglected when they conflict with the intended effects in the environment (for a review, see (Sutter et al. 2013)). However, few observations point out that individuals might shift toward a more body-centered mode of control once the incongruency becomes too pronounced (Ladwig et al. 2012; Sutter and Ladwig 2012). Our results align with the latter view: In trials where participants became aware of visuo-motor incongruency, tactile sensitivity at the operating body limb increased, presumably reflecting an upregulation of haptic processing. This resulted in more adequate compensatory movements and a tendency to produce smaller aftereffects in the next trial (e.g., (Sülzenbrück and Heuer 2011)), challenging the reasoning that performance costs posed by visuo-motor incongruency are best mitigated by neglecting body-related effects.

At the same time, our data provide only partial support for the notion that agents will downregulate incongruent body-related effects before becoming aware of their incongruency. Although participants frequently failed to notice compensatory changes to their movement, tactile sensitivity was not reduced below levels observed during congruent transformations. This suggests that controlling the action primarily via its visual effect may already be the default during congruent transformations. Only when participants realize that there has been a change in the translation of their movements into visual effects, this default appears to be overwritten, as indicated by larger radial errors preceding gain detection.

While participants did compensate for gain changes in Exp. 1, the radius of the dot movement they produced still changed substantially. Therefore, it remained possible that what participants did or did not notice when reporting a gain was a change in the visual effect rather than an incongruency with the haptic experience of their movement. Since it really was this awareness of incongruency we were interested in, we induced a higher need to compensate for the gain in Exp. 2, resulting in larger effect sizes for the tactile sensitivity comparisons. We conclude that it really is the awareness of incongruency between the agent’s felt movement and the visual effect of that movement that participants reported, rather than the detection of a visual deviation.

Our findings additionally offer a new perspective on modulations of the tactile suppression effect (for a review, see (Juravle et al. 2017)). Tactile suppression varies with temporal and spatial characteristics of the movement (e.g., (Colino et al. 2014; Juravle et al. 2010)) and decreases when the relevance of haptic action effects increases as compared to visual effects (e.g., (Colino et al. 2017; Gertz et al. 2017; Voudouris and Fiehler 2022)). Perhaps, these modulations also reflect the dominance of the respective effect domain for the control of the movement.

Altogether, we thus observed a close link between awareness of visuo-motor incongruency and haptic sensitivity at the moving limb. We tend to interpret this link as a cause-effect relationship with increased haptic sensitivity as the consequence of awareness of visuo-motor incongruency. Theoretically, other causal relationships appear also tenable: Whenever participants process haptic information more (for whatever reason), they are more likely to become aware of visuo-motor incongruency. Yet, some observations render this latter model less plausible. The detection of tactile stimulation in a given trial did not predict awareness of visuo-motor incongruency in the immediately following trial, which it should arguably do when tactile processing and awareness of visuo-motor incongruency just covaried across time, or tactile processing even facilitated the detection of visuo-motor incongruency. Conversely, there were clear aftereffects of noting visuo-motor incongruency on movement adaptation in the next trial.

Our findings underscore agents’ ability to dynamically shift between different control modes to perform the same action (Sutter and Ladwig 2012; Wirth et al. 2016) and provide promising insights into the mechanisms underlying implicit and explicit adaptation to visuo-motor conflict (e.g., (Bond and Taylor 2015; Mazzoni and Krakauer 2006; Neville and Cressman 2018; Taylor et al. 2014; Werner et al. 2015)). Given the prevalence of highly incongruent visuo-motor transformation in everyday life, this has great relevance. When operating tools with a lever, for instance, individuals immediately notice that body and tool movements do not match. Our results suggest that they respond by attending more closely to their bodily movements—at least while the transformation is still novel. Whether they revert to the default mode of controlling the action via its effects in the environment once the transformation becomes familiar is for future studies to investigate.

### Limitations

As discussed before, it may not have been awareness which caused the upregulation of body-related effect codes. Instead, fluctuations in the sensitivity to events related to the body may have increased the likelihood of detecting changes in bodily movements in response to visuo-motor incongruency. Although our experiments were not designed to test this reversed causal pathway, the data offers some preliminary evidence against it: Sensitivity to visuo-motor incongruency did not increase when participants had detected the tactile stimulus in the previous trial.

In a similar fashion, the relationship between awareness of visuo-motor incongruency and tactile sensitivity may reflect a breakdown of functional haptic neglect once incongruency becomes too large. This failure would then enable the ability to detect the adjustment to the visual gain. Crucially, however, none of our analyses supported the notion that the effect was driven by the magnitude of incongruency. Instead, the data favors the alternative model: that once participants became aware of incongruency, no matter the size, they exert top-down control over their mode of action control, attending more closely to bodily movements.

Inherent to the design was that the compensation of gains led to smaller and, critically, slower hand movements. Since movement speed has been found to relate to tactile sensitivity at the effector (Colino and Binstead 2016; (Cybulska-Klosowicz et al. 2011)), this might have contributed to differences in tactile sensitivity. Two findings suggest that this contribution was, if any, a small one: First, movement speed around the onset of tactile stimuli only predicted the odds to detect it in trials where incongruency was not detected, and not in the other conditions. Second, the large difference in speed between congruent trials and trials where incongruency was missed did not translate to a difference in tactile sensitivity.

Finally, we had to exclude a large proportion of participants because either their detection rate of the critical gain levels or of the tactile stimuli was not suitable to investigate both within the same experiment. We had anticipated and counteracted this by using gain levels successfully employed by Knoblich and Kircher (2004) in the very same paradigm and an adaptive staircase procedure to identify appropriate tactile stimulus intensities. We do not see any reason why the excluded subjects would have shown a different pattern of results, considering that they were excluded because their data did not provide enough observations to compute reliable sensitivity measures across all conditions, and not specifically based on their sensitivity in either of those conditions. Still, we encourage future studies to adopt more adaptive approaches and avoid costly exclusions: In addition to identifying appropriate stimulus intensities for each subject at the beginning, researchers should respond to changes in sensitivity online during the experiment, e.g., by using adaptive tracking procedures and compare the resulting parameters across conditions (Leek 2001). Even in designs where a trial cannot be assigned to a specific condition before its start, it might still be possible to do so within trial and before the stimulus is applied. Alternatively, adapting the stimulus’ intensity after each block of trials based on detection performance provides a means to compute signal detection theory statistics based on a single average stimulus intensity across all conditions while containing appropriate detection rates. Finally, by using a range of intensities instead of a single one, larger variations in sensitivity could be captured without changing stimulus intensities during the experiment.

## Data Availability

All experiments reported in this manuscript were preregistered on the Open Science Framework (Exp 1: https://osf.io/yqbph/overview?view_only=76f7f5ea2556469ea224344d49e1dba0, Exp 2: https://osf.io/az7ed/overview?view_only=f31e07cbe7c240b294d00c7ef720dd6b), where all materials, data, and scripts used in the experiments and their analyses are available, too (https://osf.io/nau5p/overview?view_only=bcb03444ebfe4864bb076a12e83f38cf).

## Experiment 1: average radius

In a linear mixed-effects model including those trials only, we modeled the radius at the 12 o’clock position in interval 3 (before the gain was introduced) and interval 4 (after it was introduced; (Knoblich and Kircher 2004)) as a function of gain size. The full model included gain size, gain introduction, their interaction, and random slopes for gain introduction. Accounting for gain introduction improved model fit, χ^2^(3) = 979.77, *p* < 0.001, *AIC*_*reduced*_ = 9763.4, *AIC*_*full excl interaction*_ = 8763.9, with a larger radius after the gain had been introduced, *β* = 0.58, 95% *CI* [0.53, 0.63], *t* = 21.31, *p* < 0.001. Gain size did however not explain significant variance, χ^2^(1) = 1.06, *p* = 0.303, *AIC*_*reduced*_ = 8763.0, *AIC*_*full excl interaction*_ = 8763.9, nor did the interaction, χ^2^(1) = 1.85, *p* = 0.173, AIC_reduced_ = 8663.0, *AIC*_*full*_ = 8764.1, suggesting that the radius at the 12 o’clock position of the fourth trial did not decrease significantly with increasing gain, at least across the four gain levels included in the analysis.

## Experiment 1: radial error

The radial error was computed for each frame as the absolute difference between the frame’s radius and the trial’s mean radius, either before or after gain introduction. In the first model, we tested whether the average radial error in the sixth interval was influenced by gain size and detection within the trial. The full model included gain size, gain detection, their interaction, and random slopes for gain size. Accounting for gain size significantly improved model fit, χ^2^(3) = 102.1, *p* < 0.001, *AIC*_*reduced*_ = -3871.6, *AIC*_*full excl interaction*_ = -3967.7, with larger gains predicting larger errors, *β* = 0.03, 95% *CI* [0.02, 0.03], *t* = 6.05, p < 0.001. However, neither the detection of this gain, χ^2^(1) = 0.70, *p* = 0.402, *AIC*_*reduced*_ = -3871.6, nor their interaction, χ^2^(1) = 1.76, *p* = 0.185, *AIC*_*reduced*_ = -3967.7, *AIC*_*full*_ = -3967.5, further explained any significant variance. In a second model, we scrutinized if such changes in “roundness” were perhaps not the effect of gain detection, but its cause. We computed the average radial error in the two seconds surrounding gain onset and compared it before versus after introduction, and between detected versus undetected trials. The full model included gain introduction, gain detection, their interaction, and random slopes for both. Accounting for gain introduction improved model fit, χ^2^(4) = 6227.6, *p* < 0.001, *AIC*_*reduced*_ = 4727.1, *AIC*_*full excl interaction*_ = -1492.5. The detection of gain in the trial explained further variance, χ^2^(4) = 161.8, *p* < 0.001, *AIC*_*reduced*_ = -1338.7, and so did their interaction, χ^2^(1) = 133.72, *p* < 0.001, *AIC*_*reduced*_ = -4727.1, *AIC*_*full*_ = -1624.2. In trials where the gain was detected, the radial error was smaller before the onset of gain than after, *β* = -0.34, 95% *CI* [-0.37, -0.30], *t* = -19.11, *p* < 0.001. After the gain was introduced, the radial error was smaller in trials where the gain was not detected compared to trials where it was, *β* = -0.10, 95% *CI* [-0.12, -0.09], *t* = -12.37, *p* < 0.001. The effect of gain detection was (unsurprisingly) larger after the gain had been introduced, *β* = 0.10, 95% *CI* [0.09, 0.12], *t* = 11.61, *p* < 0.001.

## Experiment 1: tactile sensitivity and gain size

We computed a linear mixed-effects model on subject-level d’ with the fixed effects gain size and gain detection. Accounting for gain detection did not significantly improve model fit, χ^2^(1) = 1.32, *p* = 0.250, *AIC*_*reduced*_ = 209.6, *AIC*_*full*_ = 209.74, but accounting for gain size did, χ^2^(1) = 6.49, *p* = 0.011, *AIC*_*reduced*_ = 214.23, *AIC*_*full*_ = 209.74. Across both detection conditions, larger gains were associated with larger d’ values, *β* = 0.06, 95% *CI* [0.03, 0.09], *t* = 3.83, *p* < 0.001. However, the model suffered from multicollinearity, VIF = 5.67, so these results should not be interpreted as evidence for an effect of gain size instead of detection.

## Experiment 2: average radius

We examined the radius at the 12 o’clock position as a function of gain size and whether it had been introduced yet, including random slopes for gain introduction. Unlike in Exp. 1, accounting for gain size improved model fit, χ^2^(1) = 16.42, *p* < 0.001, *AIC*_*reduced*_ = 5771.7, *AIC*_*full excl interaction*_ = 5757.3. Adding gain introduction further improved model fit, χ^2^(3) = 943.34, *p* < 0.001, *AIC*_*reduced*_ = 6694.6, and so did including the interaction term, χ^2^(1) = 9.30, *p* = 0.002, *AIC*_*reduced*_ = 5757.3, *AIC*_*full*_ = 5750.0. At the 12 o’clock position of the fourth interval after gain introduction, the average radius decreased with higher visual gains, *β* = -0.08, 95% *CI* [-0.11, -0.05], *t* = -5.03, *p* < 0.001. At an average gain size, it was larger at the start of the third interval before the gain change than after it, *β* = 0.73, 95% *CI* [0.67, 0.78], *t* = 26.62, p < 0.001.

## Experiment 2: radial error

We computed the radial error in each frame as the distance between the current radius and the trial’s mean radius either before or after the introduction of the gain. A first model on the average radial error in the 6th interval of a trial included the fixed effects gain size, gain detection, their interaction, and random slopes for the effect of gain size. Gain size affected the radial error, χ^2^(3) = 37.11, *p* < 0.001, *AIC*_*reduced*_ = -6072.9, *AIC*_*full excl interaction*_ = -6104.0, with larger gains predicting larger errors, *β* = 0.01, 95% *CI* [0.01, 0.02], *t* = 3.75, p < 0.001. However, neither the detection of this gain, χ^2^(1) = 2.12, *p* = 0.145, *AIC*_*reduced*_ = -6104.0, nor their interaction, χ^2^(1) = 0.13, *p* = 0.720, *AIC*_*reduced*_ = -6104.0, *AIC*_*full*_ = -6102.2, further explained any significant variance. In a second model, we scrutinized if changes in the “roundness” of the circle were perhaps not the effect of gain detection, but rather its cause. Therefore, we computed the average radial error in the two seconds around gain onset and compared it before and after it had been introduced and in trials where it was detected vs. not detected, fitting random slopes for the effect of gain introduction. The introduction of gain significantly affected the radial error, χ^2^(3) = 4572.4, *p* < 0.001, *AIC*_*reduced*_ = -1066.7, *AIC*_*full excl interaction*_ = -5633.2, and so did the detection of gain within the trial, χ^2^(1) = 346.19, *p* < 0.001, *AIC*_*reduced*_ = -5289.0. Both effects interacted significantly, χ^2^(1) = 188.99, *p* < 0.001, *AICreduced* = -5633.2, *AIC*_*full*_ = -5820.2. After the gain had been introduced, the radial error was smaller when it was not detected compared to when it was, *β* = -0.14, 95% *CI* [-0.15, -0.13], *t* = -23.26, p < 0.001. In trials where the gain was eventually detected, it was smaller before than after the gain, *β* = -0.31, 95% *CI* [-0.34, -0.28], *t* = -18.41, p < 0.001.

## Experiment 2: tactile sensitivity and gain size

We computed a linear mixed-effects model on subject-level d’ with the fixed effects gain size and gain detection. Accounting for gain detection significantly improved model fit, χ^2^(1) = 5.71, *p* = 0.017, *AIC*_*reduced*_ = 129.10, *AIC*_*full*_ = 125.39, but accounting for gain size did not, χ^2^(1) = 1.82, *p* = 0.178, *AIC*_*reduced*_ = 125.20, *AIC*_*full*_ = 125.39. At an average gain size, d’ values were lower when incongruency was not detected, *β* = -0.66, 95% *CI* [-1.19, -0.12], *t* = -2.44, *p* = 0.018. Again, the model suffered from multicollinearity, *VIF* = 6.64, and the results should be treated with caution.

See Figs. 5 and 6.

## References

[CR1] Barr DJ (2013) Random effects structure for testing interactions in linear mixed-effects models. Front Psychol 4:328. 10.3389/fpsyg.2013.00328 23761778 10.3389/fpsyg.2013.00328 PMC3672519

[CR2] Bond KM, Taylor JA (2015) Flexible explicit but rigid implicit learning in a visuomotor adaptation task. J Neurophysiol 113(10):3836–3849. 10.1152/jn.00009.2015 25855690 10.1152/jn.00009.2015 PMC4473515

[CR3] Chen J, Proctor RW (2013) Response–effect compatibility defines the natural scrolling direction. Hum Factors 55:1112–1129. 10.1177/0018720813482329 24745203 10.1177/0018720813482329

[CR4] Colino FL, Binsted G (2016) Time course of tactile gating in a reach-to-grasp and lift task. J Mot Behav 48(5):390–400. 10.1080/00222895.2015.1113917 27254788 10.1080/00222895.2015.1113917

[CR5] Colino FL, Buckingham G, Cheng DT, van Donkelaar P, Binsted G (2014) Tactile gating in a reaching and grasping task. Physiol Rep 2(3):e00267. 10.1002/phy2.267 24760521 10.1002/phy2.267 PMC4002247

[CR6] Colino FL, Lee JH, Binsted G (2017) Availability of vision and tactile gating: vision enhances tactile sensitivity. Exp Brain Res 235(1):341–348. 10.1007/s00221-016-4785-3 27722789 10.1007/s00221-016-4785-3

[CR7] Cybulska-Klosowicz A, Meftah EM, Raby M, Lemieux ML, Chapman CE (2011) A critical speed for gating of tactile detection during voluntary movement. Exp Brain Res 210(2):291–301. 10.1007/s00221-011-2632-0 21431913 10.1007/s00221-011-2632-0

[CR8] Debats NB, Heuer H (2018) Sensory integration of movements and their visual effects is not enhanced by spatial proximity. J vis 18:15. 10.1167/18.11.15 30347102 10.1167/18.11.15

[CR9] Debats NB, Ernst MO, Heuer H (2017) Kinematic cross-correlation induces sensory integration across separate objects. Eur J Neurosci 46:2826–2834. 10.1111/ejn.13758 29068094 10.1111/ejn.13758

[CR10] Dignath D, Pfister R, Eder AB, Kiesel A, Kunde W (2014) Representing the hyphen in action–effect associations: automatic acquisition and bidirectional retrieval of action–effect intervals. J Exp Psychol Learn Mem Cogn 40:1701. 10.1037/xlm0000022 24820672 10.1037/xlm0000022

[CR11] Fourneret P, Jeannerod M (1998) Limited conscious monitoring of motor performance in normal subjects. Neuropsychologia 36:1133–1140 9842759 10.1016/s0028-3932(98)00006-2

[CR12] Gertz H, Voudouris D, Fiehler K (2017) Reach-relevant somatosensory signals modulate tactile suppression. J Neurophysiol. 10.1152/jn.00052.2017 28250147 10.1152/jn.00052.2017 PMC5461667

[CR13] Hegele M, Heuer H (2010) Implicit and explicit components of dual adaptation to visuomotor rotations. Conscious Cogn 19:906–917. 10.1016/j.concog.2010.05.005 20537562 10.1016/j.concog.2010.05.005

[CR14] Heuer H, Hegele M (2008) Adaptation to visuomotor rotations in younger and older adults. Psychol Aging 23(1):190. 10.1037/0882-7974.23.1.190 18361666 10.1037/0882-7974.23.1.190

[CR15] Heuer H, Rapp K (2012) Adaptation to novel visuo-motor transformations: further evidence of functional haptic neglect. Exp Brain Res 218:129–140. 10.1007/s00221-012-3013-z 22328066 10.1007/s00221-012-3013-z

[CR16] Janczyk M, Kunde W (2020) Dual tasking from a goal perspective. Psychol Rev 127:1079–1096. 10.1037/rev0000222 32538637 10.1037/rev0000222

[CR17] Juravle G, Deubel H (2009) Action preparation enhances the processing of tactile targets. Exp Brain Res 198(2):301–311. 10.1007/s00221-009-1819-0 19407994 10.1007/s00221-009-1819-0

[CR18] Juravle G, Deubel H, Tan HZ, Spence C (2010) Changes in tactile sensitivity over the time-course of a goal-directed movement. Behav Brain Res 208(2):391–401. 10.1016/j.bbr.2009.12.009 20018212 10.1016/j.bbr.2009.12.009

[CR19] Juravle G, McGlone F, Spence C (2013) Context-dependent changes in tactile perception during movement execution. Front Psychol 4:913. 10.3389/fpsyg.2013.00913 24367346 10.3389/fpsyg.2013.00913 PMC3853591

[CR20] Juravle G, Binsted G, Spence C (2017) Tactile suppression in goal-directed movement. Psychon Bull Rev 24(4):1060–1076. 10.3758/s13423-016-1203-6 27896632 10.3758/s13423-016-1203-6

[CR21] Kadlec H (1999) Statistical properties of d’ and β estimates of signal detection theory. Psychol Methods 4:22–43. 10.1037/1082-989X.4.1.22

[CR22] Kirsch W, Pfister R, Kunde W (2016) Spatial action-effect binding. Atten Percept Psychophys 78:133–142. 10.3758/s13414-015-0997-z 26486641 10.3758/s13414-015-0997-z

[CR23] Kloss Y, Kunde W (2026) Spatially incompatible tool use does not induce tactile neglect. Atten Percept Psychophys 88:17. 10.3758/s13414-025-03170-y 10.3758/s13414-025-03170-y PMC12672823 41331181

[CR24] Knoblich G, Kircher TT (2004) Deceiving oneself about being in control: conscious detection of changes in visuomotor coupling. J Exp Psychol Hum Percept Perform 30:657–666. 10.1037/0096-1523.30.4.657 15301616 10.1037/0096-1523.30.4.657

[CR26] Kunde W, Koch I, Hoffmann J (2004) Anticipated action effects affect the selection, initiation, and execution of actions. Q J Exp Psychol A 57:87–106. 10.1080/02724980343000143 14681005 10.1080/02724980343000143

[CR27] Kunde W, Müsseler J, Heuer H (2007) Spatial compatibility effects with tool use. Hum Factors 49(4):661–670. 10.1518/001872007X215737 17702217 10.1518/001872007X215737

[CR28] Kunde W, Pfister R, Janczyk M (2012) The locus of tool-transformation costs. J Exp Psychol Hum Percept Perform 38:703. 10.1037/a0026315 22082214 10.1037/a0026315

[CR29] Ladwig S, Sutter C, Müsseler J (2012) Crosstalk between proximal and distal action effects during tool use. Z Psychol 220:10–15. 10.1027/2151-2604/a000085

[CR30] Lajoie Y, Paillard J, Teasdale N, Bard C, Fleury M, Forget R, Lamarre Y (1992) Mirror drawing in a deafferented patient and normal subjects: visuoproprioceptive conflict. Neurology 42:1104. 10.1212/WNL.42.5.1104 1579235 10.1212/wnl.42.5.1104

[CR31] Leek MR (2001) Adaptive procedures in psychophysical research. Percept Psychophys. 10.3758/BF03194543 11800457 10.3758/bf03194543

[CR32] Liesner M (2021). I control it, but does it mean it is part of me? How the relationship between body movements and controlled object movements influences the sense of agency and the sense of ownership [Doctoral dissertations, University of Wuerzburg] urn:nbn:de:bvb:20-opus-287030

[CR33] Macmillan NA (2001) Signal detection theory. In: Pashler H, Wixted J (eds) Stevens’ handbook of experimental psychology, Vol. 4. John Wiley & Sons, New York, pp 43–90

[CR34] Mazzoni P, Krakauer JW (2006) An implicit plan overrides an explicit strategy during visuomotor adaptation. J Neurosci 26(14):3642–3645. 10.1523/JNEUROSCI.5317-05.2006 16597717 10.1523/JNEUROSCI.5317-05.2006 PMC6674132

[CR35] Müsseler J, Sutter C (2009) Perceiving one’s own movements when using a tool. Conscious Cogn 18:359–365. 10.1016/j.concog.2009.02.004 19289291 10.1016/j.concog.2009.02.004

[CR36] Müsseler J, Kunde W, Gausepohl D, Heuer H (2008) Does a tool eliminate spatial compatibility effects? Eur J Cogn Psychol 20:211–231. 10.1080/09541440701275815

[CR37] Neville KM, Cressman EK (2018) The influence of awareness on explicit and implicit contributions to visuomotor adaptation over time. Exp Brain Res 236(7):2047–2059. 10.1007/s00221-018-5282-7 29744566 10.1007/s00221-018-5282-7

[CR38] Pfister R (2019) Effect-based action control with body-related effects: implications for empirical approaches to ideomotor action control. Psychol Rev 126:153–161. 10.1037/rev0000140 30604990 10.1037/rev0000140

[CR39] Pfister R, Janczyk M (2013) Confidence intervals for two sample means: calculation, interpretation, and a few simple rules. Adv Cogn Psychol 9(2):74. 10.2478/v10053-008-0133-x 23826038 10.2478/v10053-008-0133-x PMC3699740

[CR40] Pfister R, Kunde W (2013) Dissecting the response in response–effect compatibility. Exp Brain Res 224(4):647–655. 10.1007/s00221-012-3343-x 23187884 10.1007/s00221-012-3343-x

[CR41] Proctor RW, Wang DYD, Pick DF (2004) Stimulus-response compatibility with wheel-rotation responses: will an incompatible response coding be used when a compatible coding is possible? Psychon B Rev 11:841–847 10.3758/bf03196710 15732692

[CR42] Rand MK, Heuer H (2013) Implicit and explicit representations of hand position in tool use. PLoS ONE 8:e68471. 10.1371/journal.pone.0068471 23894307 10.1371/journal.pone.0068471 PMC3716878

[CR43] Rand MK, Heuer H (2018) Dissociating explicit and implicit measures of sensed hand position in tool use: effect of relative frequency of judging different objects. Attent Percept Psychophys 80:211–221. 10.3758/s13414-017-1438-y 10.3758/s13414-017-1438-y 29075991

[CR44] Sabek H, Heurley LP, Brunel L, Vanborren H, Brouillet T, Dru V (2025) Visuotactile correlation increases the integration of visual body-related effects into action representation. J Exp Psychol Human. 10.1037/xhp0001285 10.1037/xhp0001285 39964508

[CR45] Shin YK, Proctor RW, Capaldi EJ (2010) A review of contemporary ideomotor theory. Psychol Bull 136(6):943. 10.1037/a0020541 20822210 10.1037/a0020541

[CR46] Singmann H, Kellen D (2019) An introduction to mixed models for experimental psychology. In: New methods in cognitive psychology, Routledge, pp. 4–31

[CR47] Sülzenbrück S, Heuer H (2009) Functional independence of explicit and implicit motor adjustments. Conscious Cogn 18:145–159. 10.1016/j.concog.2008.12.001 19136279 10.1016/j.concog.2008.12.001

[CR48] Sülzenbrück S, Heuer H (2011) Type of visual feedback during practice influences the precision of the acquired internal model of a complex visuo-motor transformation. Ergon 54:34–46. 10.1080/00140139.2010.535023 10.1080/00140139.2010.535023 21181587

[CR49] Sutter C, Ladwig S (2012) Mirrored visual feedback limits distal effect anticipation. Exp Brain Res 218(2):247–258. 10.1007/s00221-012-3018-7 22331170 10.1007/s00221-012-3018-7

[CR50] Sutter C, Müsseler J, Bardos L, Ballagas R, Borchers J (2008) The impact of gain change on perceiving one’s own actions. In: Herczeg M, Kindsmüller MC (eds) Mensch & Computer. Oldenbourg Verlag, pp 147–156

[CR51] Sutter C, Sülzenbrück S, Rieger M, Müsseler J (2013) Limitations of distal effect anticipation when using tools. New Ideas Psychol 31:247–257. 10.1016/j.newideapsych.2012.12.001

[CR52] Taylor JA, Krakauer JW, Ivry RB (2014) Explicit and implicit contributions to learning in a sensorimotor adaptation task. J Neurosci 34(8):3023–3032. 10.1523/JNEUROSCI.3619-13.2014 24553942 10.1523/JNEUROSCI.3619-13.2014 PMC3931506

[CR53] Thébault G, Pfister R, Michalland AH, Brouillet D (2020) Flexible weighting of body-related effects in action production. Q J Exp Psychol 73:1360–1367. 10.1177/1747021820911793 10.1177/1747021820911793 32075495

[CR54] Voss M, Ingram JN, Wolpert DM, Haggard P (2008) Mere expectation to move causes attenuation of sensory signals. PLoS ONE 3(8):e2866. 10.1371/journal.pone.0002866 18682736 10.1371/journal.pone.0002866 PMC2478717

[CR55] Voudouris D, Fiehler K (2017) Enhancement and suppression of tactile signals during reaching. J Exp Psychol Hum Percept Perform. 10.1037/xhp0000373 28383966 10.1037/xhp0000373

[CR56] Voudouris D, Fiehler K (2022) The role of grasping demands on tactile suppression. Hum Mov Sci 83:102957. 10.1016/j.humov.2022.102957 35576850 10.1016/j.humov.2022.102957

[CR57] Wang L, Sutter C, Müsseler J, Dangel RJZ, Disselhorst-Klug C (2012) Perceiving one’s own limb movements with conflicting sensory feedback: the role of mode of movement control and age. Front Psychol 3:289. 10.3389/fpsyg.2012.00289 22908005 10.3389/fpsyg.2012.00289 PMC3414862

[CR58] Wendker N, Sack OS, Sutter C (2014) Visual target distance, but not visual cursor path length produces shifts in motor behavior. Front Psychol 5:25. 10.3389/fpsyg.2014.00225 24672507 10.3389/fpsyg.2014.00225 PMC3956313

[CR59] Werner S, Van Aken BC, Hulst T, Frens MA, Van Der Geest JN, Strüder HK, Donchin O (2015) Awareness of sensorimotor adaptation to visual rotations of different size. PLoS ONE 10(4):e0123321. 10.1371/journal.pone.0123321 25894396 10.1371/journal.pone.0123321 PMC4404346

[CR60] Wirth R, Pfister R, Brandes J, Kunde W (2016) Stroking me softly: body-related effects in effect-based action control. Atten Percept Psychophys 78:1755–1770. 10.3758/s13414-016-1151-2 27270736 10.3758/s13414-016-1151-2

[CR61] Sack OS, Sutter C (2017) About the role of bottom-up and top-down processes on perception-action interaction in sensorimotor transformations. J Cogn Psychol 29(4):483-496. 10.1080/20445911.2017.1279165

